# Gut Microbiota: A Potential Regulator of Neurodevelopment

**DOI:** 10.3389/fncel.2017.00025

**Published:** 2017-02-07

**Authors:** Paola Tognini

**Affiliations:** Sassone-Corsi Laboratory, Center for Epigenetics and Metabolism, Department of Biological Chemistry, University of California IrvineIrvine, CA, USA

**Keywords:** gut microbiota, neurodevelopmet, critical periods, plasticity, behavior

## Abstract

During childhood, our brain is exposed to a variety of environmental inputs that can sculpt synaptic connections and neuronal circuits, with subsequent influence on behavior and learning processes. Critical periods of neurodevelopment are windows of opportunity in which the neuronal circuits are extremely plastic and can be easily subjected to remodeling in response to experience. However, the brain is also more susceptible to aberrant stimuli that might lead to altered developmental trajectories. Intriguingly, postnatal brain development is paralleled by the maturation of the gut microbiota: the ecosystem of symbionts populating our gastro-intestinal tract. Recent discoveries have started to unveil an unexpected link between the gut microbiome and neurophysiological processes. Indeed, the commensal bacteria seem to be able to influence host behavioral outcome and neurochemistry through mechanisms which remain poorly understood. Remarkably, the efficacy of the gut flora action appears to be dependent on the timing during postnatal life at which the host gut microbes’ signals reaches the brain, suggesting the fascinating possibility of critical periods for this microbiota-driven shaping of host neuronal functions and behavior. Therefore, to understand the importance of the intestinal ecosystem’s impact on neuronal circuits functions and plasticity during development and the discovery of the involved molecular mechanisms, will pave the way to identify new and, hopefully, powerful microbiota-based therapeutic interventions for the treatment of neurodevelopmental and psychiatric diseases.

## Introduction

The development of our brain is a complicated process starting during the third week of gestation and protracting after birth, through late adolescence. Part of this process is genetically encoded, however experience and environmental stimuli play a fundamental role in refining neuronal circuits (Stiles and Jernigan, 2010). Indeed, during the postnatal period there is a remarkable remodeling of cortical and subcortical structures. In particular, the neuronal circuits belonging to sensory systems encounter an important experience-dependent reshaping.

The brain circuitry is particularly plastic to environmental inputs during preferred windows of opportunity called sensitive periods (Berardi et al., 2000). Furthermore, critical periods are special sensory periods, in which the influence of experience results in irreversible brain changes (Knudsen, 2004). Critical periods have been demonstrated for many species, from *Drosophila* to rodents and humans and for different functions. Interestingly, the opening of some specific critical periods in mice coincides with weaning. For example, the critical period for ocular dominance plasticity opens at around P21–23 in mice that perfectly matches with the passage from breast milk to a solid diet. Remarkably, it has been demonstrated that in humans the cessation of breast-feeding is one of the major determinants for the assembly of an adult microbiome (Bäckhed et al., 2015) and that diet can rapidly alter the composition of commensal bacteria (David et al., 2014). In the last decade, the gut microflora has raised the interest of the scientific community and many studies have begun to investigate its role on different aspects of host physiology. The gut microbiota has been implicated in the development of the host immune system and in shaping its responses during infectious diseases (Round and Mazmanian, 2009; Brestoff and Artis, 2013). Moreover, commensal bacteria are instrumental in the control of host energy metabolism and metabolic homeostasis (Tremaroli and Bäckhed, 2012).

Recently the gut microbiota has been involved in behavioral modulation in both rodents and humans (Foster and McVey Neufeld, 2013), suggesting the intriguing possibility of the microbiota’s influence on brain development and function. So far, the majority of studies have focused on the impact of microbes on the adult central nervous system (CNS). However, some evidence seems to indicate that the microbiota might also be involved in the development of neuronal circuits, and that a healthy microbiome early in life might play a key role for a correct neurodevelopment (Borre et al., 2014). As stated, breast-feeding cessation is important for microbiome maturity, while during the same period the brain faces a dramatic remodeling at circuit and synaptic levels. Therefore, the microbiota may somehow affect and contribute to the process of neuronal circuitry refinement during postnatal development.

This review article will focus principally on studies investigating the crosstalk between the brain and gut microbiome, and how the microbiota seems to affect neuronal function and consequently, the behaviors of the host. Finally, recent findings linking the commensal bacteria to neurodevelopmental disorders will be discussed.

## Gut Microbiota Maturation

Our body has a longstanding partnership with a myriad of microorganisms, such as bacteria, viruses, protozoa, archaea, fungi, that constitute the so-called microbiota (Sekirov et al., 2010). These symbionts colonize all the body surfaces that are in contact with the external environment from birth and arguably *in utero*, establishing a lifelong relationship with their host. Bacteria are the best studied and they seem to be particularly enriched in the gastrointestinal (GI) tract. The gut microbiota comprises trillions of bacteria, characterized by approximately 1000 species and outnumber the genes present in the human genome (Ley et al., 2006; Qin et al., 2010). Indeed the intestinal microbiome has been defined as a “forgotten organ” (O’Hara and Shanahan, 2006). The gut ecosystem is dominated by two bacterial phylotypes: Firmicutes and Bacteriodites, and as established by metagenomic sequencing, besides inter-individual variability, the core gut microbiota and microbiome are shared in humans (Qin et al., 2010). The intestinal bacteria behave like a factory and produce fundamental molecules for many host biological processes, such as vitamins, short chain fatty acids (SCFA: propionate, acetate and butyrate), amino acids and their derivatives and, interestingly, neurotransmitters. Moreover, they participate in the metabolism of choline, bile acids, polyamine and various lipids (Nicholson et al., 2012).

Colonization of the human GI tract begins at birth. In fact, the fetus was thought to be completely sterile until few years ago when provocative studies demonstrated the presence of bacteria in the placenta and amniotic fluid (Jiménez et al., 2008; Aagaard et al., 2014; Collado et al., 2016). During vaginal delivery, the baby is exposed to microbes mainly belonging to the mother’s vaginal tract and feces; although the bacteria present in the external milieu, such as the hospital in developed countries, also contribute to the initial colonization of the babies’ intestine. C-section strongly influences the neonates’ microbiota that resembles predominantly the one of the mother’s skin (Matamoros et al., 2013). Thus, the mode of delivery, the type of feeding (breast or formula milk), the mother’s diet, the environment and the use of antibiotics significantly impact the composition of the neonatal gut microbiota and affects its maturation later in life.

The passage from a milk-based diet to solid food is extremely important in the maturation of the gut flora (Jiménez et al., 2008; Aagaard et al., 2014; Collado et al., 2016) and in humans, the first 3 years of life seems to be critical for the assembly of the commensals ecosystem. At the same time, intense synaptogenesis occurs in the brain (Tau and Peterson, 2010). Notably, the refinement of neuronal circuits protracts during adolescence, in which a dramatic synaptic pruning takes place, and during adulthood, when, although not at the same extent, some synaptic rewiring is still present (Petanjek et al., 2011). In parallel, the microbiota evolves from childhood/adolescence, characterized by less diversity between species (Agans et al., 2011), to adult age. In adulthood, the gut microbial ecosystem reaches its maturity, becomes more diverse and stable overtime and more resistant to perturbation (i.e., antibiotic use, dietary changes, stress; Rajilić-Stojanović et al., 2013). It is important to note that postnatal neurodevelopment and gut microbiota evolution co-occur, suggesting the intriguing possibility of a bidirectional influence between brain and commensal bacteria on each other’s maturation.

## The Gut Microbiota Sculpts Brain Function and Behavior

Can postnatal microbioal colonization act and have a long-lasting effect on host brain physiology and plasticity? A ground-breaking study from 2004 paved the way to the new concept of gut microbiota impact on brain function and behavior. Sudo et al. (2004) demonstrated that germ free (GF) mice (completely sterile animals that have never had contact with any microorganism) displayed an exaggerated stress response with respect to specific pathogen free (SPF) mice, animals raised with a normal gut flora. The behavioral phenotype was accompanied by decreased expression of cortical and hippocampal brain derived neurotrophic factor (BDNF), a neurotrophin involved in neuronal survival, differentiation, growth, and synaptic plasticity (Park and Poo, 2013). The oral administration to GF mice of a single strain of bacterium, *Bifidobacterium infantis*, rescued the HPA stress response. Intriguingly, the fecal transplant of SPF mice microbiome to GF mice at an early developmental stage successfully restored the stress response to basal levels, although the same procedure was not efficacious at later age (Sudo et al., 2004). Similarly, reduced anxiety-like behavior and increased locomotor activity in GF mice could be reverted by conventionalization with SPF microbiota only in young but not in adult mice, suggesting the presence of a period of opportunity for the action of the gut microbes on neuronal circuits function and plasticity. Remarkably, the hippocampus, frontal cortex and striatum of GF mice had dramatic differences in gene expression with respect to SPF. Moreover, synaptophysin, a protein important for synaptic vesicles endocytosis and synaptogenesis (Tarsa and Goda, 2002; Kwon and Chapman, 2011), and PSD95, involved in excitatory synaptic maturation and plasticity (El-Husseini et al., 2000; Béïque and Andrade, 2003), were reduced in the striatum of GF mice (Diaz Heijtz et al., 2011), indicating that alteration in synaptic plasticity could be linked to the absence of the intestinal commensals. Clarke et al., found that the gut microbiome influences the development of the hippocampal serotonergic system. Indeed, serotonin levels in the hippocampus of GF mice were increased and correlated with reduced anxiety, although no direct link was demonstrated between the neurotransmitter change and the behavioral phenotype. Post-weaning colonization was not able to restore the hippocampal neurochemical differences with respect to the conventionalized mice, however, it could revert the altered anxiety-like behavior (Clarke et al., 2013). Modifications in plasticity related genes were also noticed in the amygdala of GF mice, in which a significant down-regulation of the NR2B subunit of the NMDA receptor, important for synaptic plasticity and memory process, was observed and paralleled by reduced anxiety-like behavior (Neufeld et al., 2011). Remarkably, the total absence of microbes in GF mice was accompanied by an enlargement in the amygdala and hippocampal volume, notwithstanding the whole brain volume was the same. Furthermore, area-specific alteration in dendritic spines density and morphology, and dendrites’ length were reported. The structural modifications might correlate with the behavioral profile of GF animals, however no causal evidence has been demonstrated (Luczynski et al., 2016). Moreover, the analysis was performed only in adult mice leaving an open interrogative about when early in life these morphological changes occur and how they could affect developmental trajectories. Antibiotic treatment of SPF rodents from weaning, in order to ablate the intestinal commensals, had a strong impact on brain chemistry and behavioral outcomes resulting in anxiolytic effects, memory impairments and deficit in the social transmission of food preference test (Desbonnet et al., 2015). This study emphasizes the importance of the microbial ecosystem influence on neuronal physiology during crucial periods of development such as the post-weaning, which is comparable to adolescence in humans. Even in adult mice, a short-term broad-spectrum antibiotic administration could decrease anxiety and up-regulate BDNF expression in the hippocampus (Bercik et al., 2011). It is worth noting that the depletion of the gut microbiota, either through antimicrobial treatment or GF conditions, results in anxiolytic outcomes apparently in conflict with the increase in stress reactivity of GF mice (Sudo et al., 2004; Clarke et al., 2013). It is difficult to explain the discrepancy, especially considering the complexity of the experimental designs in the different studies; however this discordance features the possible intricate and not yet unveiled effects of the microbial community or individual bacteria on the host brain physiology during development and adulthood, opening challenging questions for the future.

As highlighted, neuroplasticity is a complicated phenomenon, highly prominent in juvenile ages. Nevertheless, the adult brain can, at some extent, dynamically reorganize its structure by modulating synaptic connection between neurons. Fascinating, newly born neurons are produced in specific area of the adult CNS, and they have been involved in learning and memory processes. Adult neurogenesis is sensitive to a variety of environmental stimuli such as stress (Mirescu and Gould, 2006) and, intriguingly, the gut microbiota has been shown to be one of them. Indeed, dorsal hippocampal neurogenesis was higher in adult GF mice compared to control animals. Colonization of young GF mice at weaning could not normalize the phenotype (Ogbonnaya et al., 2015), underscoring the possibility that different neurophysiological processes could be fine-tuned by the signals from the intestinal microbes during specific sensitive periods, and that adult neurogenesis could be primed by microbiome cues early after birth. Furthermore, long-term antibiotic administration in adult SPF mice significantly decreased hippocampal neurogenesis, although it could be completely restored by probiotics or voluntary exercise. An interesting mechanistic explanation for the reduced neurogenesis upon antimicrobial treatment is the decreased infiltration of specific immune cells: Ly6C^hi^ monocytes. Ly6C^hi^ population was rescued by probiotics and exercise and strikingly, the adoptive transfer of Ly6C^hi^ monocytes in animals treated with antibiotics fully rescued hippocampal neurogenesis (Möhle et al., 2016). This discovery sheds light on the link between immune cells, brain plasticity and gut microbiome pointing to the immune system as a route to transduce the commensal bacteria action on the CNS.

The discussed evidence suggests that the intestinal microbiota could contribute to the shaping of neural networks during development, affecting behavior and neuronal physiology later in life. Indeed, we might speculate that together with inborn genetic factors and novel experience inputs, the bacteria derived signals could act on an immature and plastic postnatal brain substrate to fine-tune further remodeling of synaptic connections and neuronal circuitry. However, so far no direct evidence of an action of the gut microbiome on specific neuronal populations function, or on the physiology of brain circuits especially during neurodevelopment has been demonstrated. The bacterial ecosystem and its perturbation are associated with certain neurochemical, structural changes and/or behavioral outcomes, mainly analyzed in adult animals. It is unclear if the presence, absence, or perturbation of the microbiome community is causative and promotes the molecular and phenotype alterations, or if the modifications are a consequence of secondary effects. The lack of mechanistic insights resides, at least in part, on the complexity of the communication routes between the intestinal ecosystem and the CNS, which integrates neuronal, hormonal and immunological signaling. In the next section an overview of the possible dual cross-talk pathways connecting brain and intestine will be examined.

## GI Tract-Brain Crosstalk Pathways and Their Relevance in CNS Function

### Enteric Nervous System and Vagus Nerve

A well-known path is represented by the vagus nerve. The gut microbes could directly activate the enteric nervous system (ENS) that innervate the entire length of the microvilli (Furness, 2012), and through the vagus nerve transmit inputs to the brain. Indeed, vagotomy prevented the anxiolytic and antidepressant effect of a probiotic treatment (*Lactobacillus Rhamnosus* (JB-1)) in conventionally raised BALB/c mice, and completely inhibited the modifications in GABA_A_ receptor gene expression in hippocampus and amygdala (Bravo et al., 2011). However, no functional changes in neuronal circuits of these specific areas were investigated, opening further questions on how the stimulus from the probiotic administration through the vagus nerve could be translated in the behavioral phenotype. Notably, some of the behavioral traits affected by the intestinal commensals are not dependent on the vagus nerve and the autonomic nervous system. In fact, the administration of an antibiotic cocktail was able to perturb the microbiome composition, increase exploratory phenotype and expression of BDNF in the hippocampus of SPF mice, and strikingly, these effects did not rely on an intact vagal connection (Bercik et al., 2011). These findings underlie the intricate crosstalk between intestinal flora and CNS, suggesting multiple and mostly unexplained lines of actions of the gut symbionts on complex behaviors and neurophysiology.

### Circulation

The intestinal epithelium regulates the passage of nutrients and other substances into the blood stream. A variety of metabolites and neurotransmitters are produced by the gut microorganisms, enabling a complicate exchange of sensory information with the host (Sharon et al., 2014). The microbes secrete vitamins, SCFA, serotonin, GABA, acethylcoline, dopamine and noradrenaline etc. into the gut lumen. After absorption and via the circulatory system, some of these molecules can eventually cross the blood-brain barrier (BBB) and reach the brain in which they could play active functions. How these substances can influence the physiology of neuronal circuits is still unknown. Among the various metabolites, SCFA, the end products of complex-polysaccharide fermentation, have been involved in metabolic homeostasis, infection and inflammation, host-microbiota interactions etc. (Ríos-Covián et al., 2016). They are up-taken by glial cells and neurons in the CNS and exploited as an energy source, especially in the developing brain (Rafiki et al., 2003). Interestingly, butyrate and propionate act as histone deacetylase (HDAC) inhibitor (Waldecker et al., 2008) and they might influence gene expression and the host epigenome through chromatin remodeling in various tissues, including the brain.

### Interaction through the Immune System

The intestinal symbionts are fundamental for the development of the host immune system and this seems to be a key indirect communication route between the gut microbiota and the CNS. The gut flora produces microbial-associated molecular patterns (MAMPS) and metabolites that can stimulate immune cells’ function and eventually influence neurophysiology and behavior (Mu et al., 2016). Remarkably, the gut microbiome is crucial for the maturation of microglial cells, the tissue resident macrophage of the brain. Depletion of the intestinal commensal dramatically jeopardized microglia development and function in adult mice, that maintained a more juvenile state and decreased its responses to MMPA and pathogen encounter. Strikingly, the administration of SCFA was able to completely revert the impairment, indicating that signals from the gut flora play an essential role in microglia function maintenance (Erny et al., 2015). It is worth noting that microglia are emerging as a key players in synaptic pruning in the developing brain, consisting in the elimination of select synapses to ensure a correct wiring of the neuronal network (Paolicelli et al., 2011). Furthermore, even in absence of an inflammatory process, microglial cells release immune-related signaling molecules, which can impact neuronal transmission and synaptic plasticity (Wu et al., 2015). Based on this evidence, we could hypothesize that during neurodevelopment the intestinal microbiota might fine-tune neuronal circuits function and plasticity via the modulation of microglial cell action.

Different types of signals generated by the GI tract microbiota might directly reach the host brain or be translated to the CNS in an indirect fashion soon after birth and during sensitive periods for neurodevelopment. Thus, aberrant alteration in the commensal composition (e.g., antibiotics, mode of delivery, stress, diet) could deteriorate gut-brain communication pathways and be partially responsible for an abnormal shaping of host neuronal circuits and, consequently, for the behavioral impairment occurring in neurological diseases.

## Contribution of the Intestinal Microbiota to Altered Neurodevelopmental Trajectory

New epidemiological and clinical evidence has shown comorbidity between neurological illness and GI tract disturbances. For instance, a subset of autism-spectrum disorders (ASD) patients exhibit GI symptoms, abdominal pain, increased intestinal permeability (Valicenti-McDermott et al., 2006; Boukthir et al., 2010; de Magistris et al., 2010; Coury et al., 2012; Hsiao, 2014) and, intriguingly, an altered composition of their gut microbiome (Kohane et al., 2012; Kang et al., 2013; Wang et al., 2013), implying a possible link between dysbiosis (an aberrant shift in microbial ecology) and neurodevelopmental diseases. Autism is an extremely heterogeneous neurodevelopmental disorder and its onset can vary, with some children showing signs from early infancy while others exhibit symptoms of regression at the age of 2–3 years. Notably, during the second and third year of life the microbiome is subjected to an important evolution, becoming more similar to the adult ecosystem (Yatsunenko et al., 2012), indicating a further link between commensal bacteria and ASD. Autism is characterized by stereotypical behaviors, and deficit in social interactions and communication (Wang and Doering, 2015). Intriguingly, GF mice displayed social skills impairment and excessive self-grooming, considered equivalent to repetitive behaviors in humans. Post-weaning colonization partially reversed the phenotype, ameliorating self-grooming, social avoidance but not social cognition (Desbonnet et al., 2014), indicating that the window of opportunity for the microbiota to impact brain circuits might be different for distinct emotional/social behaviors and, eventually, sensory modalities.

Antibiotic use during pregnancy is a common practice (Broe et al., 2014), although little is known about their effect on the fetus development. Perinatal antibiotic treatment in pregnant mice disrupted the microbiome population of both mother and offspring. Remarkably, alteration in the offspring behavior characterized by decreased locomotor and explorative activity was observed at 4 weeks of age, a phenotype that was completely reverted by fostering the pups by control dams (Tochitani et al., 2016). Similarly, periconceptional succynilsulfathiazole (a non-absorbable antibiotic) exposure in rat dams significantly modified the progeny’s performance in several tests. Indeed, the offspring exhibited low social interactions, higher anxiety, a deficit in prepulse inhibition, which are behavioral abnormalities associated with ASD, schizophrenia and other human mental disorders (Degroote et al., 2016). Despite the lack of mechanistic insights, these works highlight the importance of a healthy mother microbiota to circumvent potential abnormal behavioral trajectories in the offspring.

As already mentioned, the dietary regimen of the mother is an important element in the establishment of the neonates’ intestinal ecosystem. Maternal obesity has been connected to an increase in the risk of neurodevelopmental illness and ASD incidence in children (Krakowiak et al., 2012; Sullivan et al., 2014; Connolly et al., 2016). A very new and insightful study in rodents has revealed a possible mechanism. The offspring of high fat diet fed mice displayed social deficits driven by gut microbiome dysbiosis. In fact, the co-housing and subsequent microbiota sharing with the offspring of lean mothers, restored the social impairments. Notably, a single probiotic species, *Lactobacillus reuteri*, could ameliorate the social skills and rescue impaired long-term potentiation in the ventral tegmental area (Buffington et al., 2016). Another probiotic, *Bacteriodes fragilis*, has been demonstrated to be beneficial in mice showing ASD-related symptoms and dysbiosis. In particular, *B. Fragilis* was able to remodel the gut microbiome and to improve communication, repetitive and anxiety-like behaviors in mice, however it could not correct social abilities (Hsiao et al., 2013).

Increasing evidence supports the possibility that other neuropsychiatric disorders of potential neurodevelopmental origins, such as schizophrenia (Dinan et al., 2014; Sherwin et al., 2016), bipolar disorder (Dickerson et al., 2016), depression (Hagan et al., 2015; Mangiola et al., 2016), might be dependent on the microbiome composition early in life. Focusing on schizophrenia, premature birth, that is associated with microbiota changes (Groer et al., 2014; Gibson et al., 2016), positively correlates with schizophrenia incidence (Johnson and Marlow, 2011; Nosarti et al., 2012). Furthermore, alteration of the immune system (Dickerson et al., 2016) and impaired GI functions are frequently present in psychotic patients (Severance et al., 2016). Unfortunately, no information is available on the fecal microbiota composition in individuals affected by schizophrenia; nonetheless the oropharyngeal microbiome in adult schizophrenic subjects exhibited significant differences at the phylum and species levels with respect to non-psychiatric controls (Castro-Nallar et al., 2015). In rodents, there are no systematic studies investigating the role of the intestinal microbes in schizophrenia. However, an interesting work demonstrated that the microbiota contributes to the myelinization process specifically in the prefrontal cortex (PFC): a brain region involved in attention, memory, emotional learning, and critically connected to neuronal disorders such as ASD and schizophrenia. GF mice displayed an upregulation in the gene program linked to myelinization, and hypermylinated axons in the PFC. Importantly, post-weaning bacteria colonization was not able to normalize myelin oligodendrocyte glycoprotein (MOG) levels (Hoban et al., 2016), again pointing toward the existence of specific critical periods for the microbiota actions on the brain. These results reveal new avenues to better understand the possible relationship between gut microbiome and myelin maturation, and subsequently its impact on the onset of psychiatric diseases linked to myelinization abnormalities.

Although the link between dysbiosis, feeding habits, periconceptional/perinatal antibiotic use and behavioral abnormalities in neurodevelopmental disorders has still to be clarified, based on these studies and clinical reports (Critchfield et al., 2011; Fond et al., 2015), probiotics, prebiotics and dietary manipulations in early childhood may represent promising interventions for ameliorating some autistic features and, eventually, for preventing an increase in the incidence of ASD or other neuropsychiatric illnesses.

## Future Outlooks

The last two decades have been characterized by exciting discoveries unveiling an unexpected link between intestinal flora and brain function. PFC myelinization (Gacias et al., 2016; Hoban et al., 2016), behavioral responses to drugs of abuse (i.e., cocaine; Kiraly et al., 2016), integrity of BBB (Braniste et al., 2014), ischemic brain injury (Benakis et al., 2016) are just few of the phenomena (see also previous sections) that have been demonstrated to be affected by commensal bacteria. Furthermore, the gut microbes seem to be an environmental factor impacting brain development possibly through metabolites or other microbiota-derived dependent molecules, subsequently influencing behavior later in life. The fascinating observation of a critical period in which the microbiota could affect neurodevelopment and the existence of sensitive periods for experience-dependent refinement of sensory modalities (i.e., vision, hearing etc.), language acquisition, fear extinction and many others processes, suggests the appealing possibility of microbiome-driven stimuli involvement in fine-tuning neuronal circuits remodeling during early age. Although some studies have shown microbiota-dependent modifications in brain chemistry and behaviors, there are no specific molecular mechanisms explaining how neurophysiology can be altered by the microbiome and how changes in the gut commensals during critical periods of postnatal neurodevelopment might impact neurodevelopmental trajectories, eventually resulting in ASD and/or in other neurological diseases. Indeed, to dissect the mechanisms underlying the gut microbiome actions on the brain during sensitive periods and, on the other hand, how signals from the CNS can shape the microbiome ecosystem, will reveal new avenues of future directions to apply microbiota-based therapeutics for the treatment of neurodevelopmental, psychiatric and in general neurological disorders, highly pervasive illness in the modern society.

## Author Contributions

PT conceived and wrote the review article.

## Conflict of Interest Statement

The author declares that the research was conducted in the absence of any commercial or financial relationships that could be construed as a potential conflict of interest.
